# The uptake and use of a minimum data set (MDS) for older people living and dying in care homes: a realist review

**DOI:** 10.1186/s12877-021-02705-w

**Published:** 2022-01-07

**Authors:** Massirfufulay Kpehe Musa, Gizdem Akdur, Sarah Brand, Anne Killett, Karen Spilsbury, Guy Peryer, Jennifer Kirsty Burton, Adam Lee Gordon, Barbara Hanratty, Ann-Marie Towers, Lisa Irvine, Sarah Kelly, Liz Jones, Julienne Meyer, Claire Goodman

**Affiliations:** 1 Centre for Research in Public health and Community Care (CRIPACC), School of Health and Social Work, University of Hertfordshire, Hatfield, UK; 2 National Institute for Health Research Applied Research Collaboration South West Peninsula (PenARC), University of Exeter Medical School, St Luke’s Campus, Heavitree Road, Exeter, UK; 3 Faculty of Medicine and Health Sciences, University of East Anglia, Norwich, UK; 4 School of Healthcare, University of Leeds, Leeds, UK; 5 NIHR Applied Research Collaboration, Yorkshire and Humber, Leeds, UK; 6 Institute of Cardiovascular and Medical Sciences, University of Glasgow, Glasgow, UK; 7 School of Medicine, University of Nottingham, Nottingham, UK; 8 NIHR Applied Research Collaboration, East Midlands (ARC-EM), Leicester, UK; 9 Population Health Sciences Institute, Campus for Ageing and Vitality, Newcastle University, Newcastle upon Tyne, UK; 10 NIHR Applied Research Collaboration, North East and North Cumbria, Newcastle, UK; 11 Centre for Health Services Studies, University of Kent, Canterbury, UK; 12 NIHR Applied Research Collaboration, Surrey and Sussex, Kent, UK; 13 Cambridge Public Health, University of Cambridge, Cambridge, UK; 14 National Care Forum, Friars House, Manor House Drive, Coventry, UK; 15 Care for Older People, School of Health Sciences, Division of Nursing, City, University of London, London, UK; 16 NIHR Applied Research Collaboration East of England, Cambridge, UK

**Keywords:** Older people care, long-term care, care home, standardised care, minimum-data-set

## Abstract

**Background:**

Care homes provide long term care for older people. Countries with standardised approaches to residents’ assessment, care planning and review (known as minimum data sets (MDS)) use the aggregate data to guide resource allocation, monitor quality, and for research. Less is known about how an MDS affects how staff assess, provide and review residents’ everyday care. The review aimed to develop a theory-driven understanding of how care home staff can effectively implement and use MDS to plan and deliver care for residents.

**Methods:**

The realist review was organised according to RAMESES (Realist And Meta-narrative Evidence Synthesis: and Evolving Standards) guidelines. There were three overlapping stages: 1) defining the scope of the review and theory development on the use of minimum data set 2) testing and refining candidate programme theories through iterative literature searches and stakeholders’ consultations as well as discussion among the research team; and 3) data synthesis from stages 1 and 2. The following databases were used MEDLINE via OVID, Embase, CINAHL (Cumulative Index to Nursing and Allied Health Literature), ASSIA [Applied Social Sciences Citation Index and Abstracts]) and sources of grey literature.

**Results:**

Fifty-one papers informed the development of three key interlinked theoretical propositions: motivation (mandates and incentives for Minimum Data Set completion); frontline staff monitoring (when Minimum Data Set completion is built into the working practices of the care home); and embedded recording systems (Minimum Data Set recording system is integral to collecting residents’ data). By valuing the contributions of staff and building on existing ways of working, the uptake and use of an MDS could enable all staff to learn with and from each other about what is important for residents’ care

**Conclusions:**

Minimum Data Sets provides commissioners service providers and researchers with standardised information useful for commissioning planning and analysis. For it to be equally useful for care home staff it requires key activities that address the staff experiences of care, their work with others and the use of digital technology.

**Registration:**

PROSPERO registration number CRD42020171323.

**Supplementary Information:**

The online version contains supplementary material available at 10.1186/s12877-021-02705-w.

## Background

In the UK, an estimated 1.6 million people are aged 85 years and above [1]. Longevity of the oldest old (age 85 years and above), is associated with higher levels of dependency and projected need for long term care [2]. About 420,000 older people in England and Wales live in care homes [3, 4]. Care home is a generic term referring to facilities in which people live together with staff on-site 24 hours a day to provide care, with some homes having on-site registered nurses [5]. Whilst care homes may provide short-term respite care, for most people they are the sole place of residence or home. The care home population encompasses some of the most vulnerable members of society, with approximately 70% living with cognitive impairment [6, 7]. The health and care needs of this population are met by a range of health and social care staff working in, or with, care homes. Information about residents’ characteristics, needs and services they receive sits in multiple unaligned health and social care databases. Without a national core dataset based on resident-level information very little, outside of research evidence is known about this population [8]. An increasing post pandemic priority is ensuring efficient and effective sharing of resident data for the purposes of care, planning and evaluating services.

The COVID-19 pandemic in the UK highlighted the consequences of having no nationally mandated data collection on care home residents or links with National Health Service (NHS) records [8–10]. Not having standardised and accessible information about care home residents’ medical history, service use and care needs, had a negative impact on the public health response for this population [11]. This delayed recognition of excess mortality in care homes and policy measures that were care home specific for infection prevention strategies for residents and staff [12]. All care homes collect substantial amounts of data about their residents and many use validated assessments, for example for nutritional status, falls risk and dependency levels [13].The need for a common approach to data capture or links with health care data sets is a policy priority and an implementation challenge [14],

There are different versions of MDS used in long term care around the world (for example the MDS 3.0 ( Saliba and Buchanan )[15] used in USA and International Resident Assessment Instrument (Inter-RAI) used extensively in Canada and adapted for different care systems in New Zealand, parts of Australia and some countries of mainland Europe [16], . The use of an MDS is often but not always mandated and/or linked to national reimbursement. For example, in the USA Medicare reimbursement is based on responses from the MDS 3.0 to determine residents’ care needs. MDS support comprehensive assessment of care home residents and linked care planning, enable multidisciplinary working, quality assessment, and inform commissioning of services [17–24]. There are administrative costs and concerns about the burden that they place on staff, the depersonalisation of care and if consistency across care systems can be achieved [25, 26].

Previous research in the UK has tested existing standardised approaches to resident assessment and data [27, 28]. but there is limited work on what needs to be in place for effective implementation. The starting assumption of this realist review is that decision-making for care home residents’ care can be enhanced through the application of data that can be used by a range of stakeholders [29]. The review’s particular focus is on how long-term care settings make the transition to standardised approaches to data collection, and how its use impacts on staff work, time away from care, knowledge of the care home residents, working with other healthcare professionals.

An MDS is defined as a standardised account of the demographic, social, and health characteristics and needs of older people living in long-term care (care home) settings.

### Aim

To develop a theory-driven understanding of how care home staff can effectively implement and use MDS to plan and deliver care for residents.

#### Objectives

1. Develop a programme theory describing contexts that support the uptake and use of an MDS in care homes.
2. Identify in what circumstances the use of an MDS produces improved outcomes (including resource use) for an individual resident, their family, and the care home staff and their employing organisation.

## Methods

A realist review to develop a theory of what needs to be in place for effective MDS uptake and use at the resident level of care [30–33]. This theory-driven approach to reviewing research evidence on complex social interventions can provide an explanatory analysis of how and why interventions work (or not) in particular contexts or settings, as well as unintended consequences [32, 34].

Realism asserts that it is not interventions that create change; rather, it is the people involved and their responses [32, 35, 36]. This review uses the evidence to identify and test the interactions between contexts, mechanisms and outcomes (or ‘CMOs’), to provide an explanatory account of how an intervention works (Table 1).

**Table 1:** Glossary of realist terms in this review

**Contexts (C)** – Are often ‘the ‘backdrop’ of programmes and research…broadly understood as any condition that triggers and/or modifies the behaviour of a mechanism [39].	
**Mechanisms (M)** – are not observed directly but account for what it is about programmes that makes them work, characterised as “underlying entities, processes or structures which operate in particular contexts to generate outcomes of interest” ([40], p.368). Mechanisms are the responses of those involved in an intervention/programme to the resources or opportunities offered by that intervention/programme. Responses may include thoughts, feelings or actions. They are activated or inhibited by circumstances or contexts that then have an effect ([31], p.xvii).	
**Outcomes (O)** – are strategies of the intervention/programme (planned or unplanned, visible or not); result of the interaction between a mechanism and its triggering context [31, 41].	
**Programme theories** – an overarching theory or model of how a programme, or an intervention is expected to work, and it helps to explain (some of) ‘how and why, in the “real world”, a specific programme “works”, for whom, to what extent and in which contexts’ [37, 40].	
**Demi-regularities** – a “prominent recurrent patterns of contexts and outcomes… in the data” ([33], p. 9).	
**Context-Mechanism-Outcome (CMO)** – CMO an heuristic used to explain generative causation, which help to explain the relationship between a context, mechanism, and an outcome of interest in a particular programme [42]. It demonstrates the causal components that explain what works in an intervention/programme for who, why and in which circumstances [31].	

This review draws on practical ‘how-to’ guidance [34, 37], and follows publication standards (Realist And Meta-narrative Evidence Syntheses: Evolving Standards (RAMESES)) guidance [32]. A more detailed account of the methods is published elsewhere [38].

**Fig. 1:**
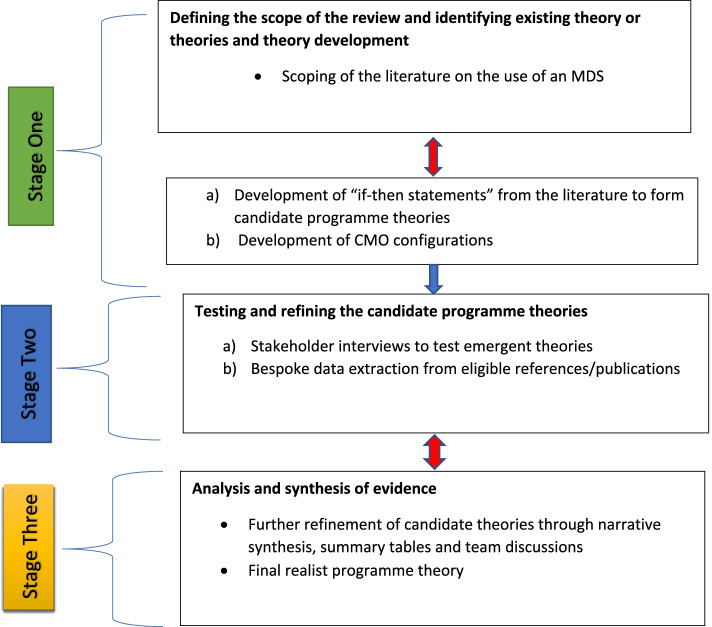
The three-staged approach to the synthesis

Realist reviews go beyond identification of barriers and facilitators to provide a theory-driven explanation of what needs to be in place for implementation [30–32]. The review is organised in three stages (Fig. 1). First, a scoping of the literature to identify care home specific work on the acceptance and use of MDS in care home settings. Next, a theory driven review of the evidence, plus interviews with key stakeholders to test and refine theories that explain the use of an MDS and linked resident and staff outcomes. Finally, a synthesis of the evidence to establish how and when the use of an MDS achieves different outcomes for residents, families, staff, and organisations and presentation of a final programme theory.

## Changes from the submitted protocol in the review process

Realist review is an iterative process; thus, adjustments were made to the review protocol [38] in the light of emerging or new lines of enquiry. The intention had been at the beginning of the review to conduct eight interviews with key stakeholders with experience of implementing and using MDS for care work. There were changes to the focus and timing of interviews to explore in more detail how staff engaged with new ways of capturing data and allow a greater emphasis on whether findings resonated with those of the interviewees. Stakeholders’ availability and recruitment was affected by the Covid-19 pandemic [43]. Four theory-driven interviews were completed (care home manager, care home staff member, international researcher with experience of implementing MDS and care home board member) (Online supplementary 1). Consequently, more time was given to drawing on the experiences and knowledge of the wider research team and study steering group of what supports data collection and staffs’ use of standardised measures in care homes. The study steering group membership included three leaders of care home organisations and their representative bodies, two resident representatives, three care home researchers, a clinician working with care homes and three long term care data specialists.

Ethical approval for interviews was received from the University of Hertfordshire Ethics committee (HSK/SF/UH/04169).

All methods were performed in accordance with the relevant guidelines and regulations.

### Stage 1: Defining the scope of the review, identifying existing theories (concept mining and candidate theory (theories) development)

The exploratory scoping of the literature began with evidence nested within a larger review on assessment and outcome measure used in care home research (PROSPERO reference: CRD42020155923). Between January to July 2020, we searched bibliographic databases MEDLINE via OVID, Embase, CINAHL (Cumulative Index to Nursing and Allied Health Literature), ASSIA [Applied Social Sciences Citation Index and Abstracts]) and sources of grey literature. We included literature published in English language over the last ten years for and applied terms such as care homes, skilled nursing facilities, long-term care facilities, and nursing homes, and then combined those terms with others such as MDS, inter-RAI, Geriatric Assessment, and Research Assessment Instrument (Online supplementary 2).

The review drew on evidence from a wide range of sources [30, 35]. A paper was included if the evidence was sufficiently detailed to be assessed “*good enough and relevant*” implementation and use of MDS in care homes [38, 44].

The data extraction and quality appraisal of included documents were done simultaneously [45]. A series of ‘if-then’ statements based on the evidence (Online supplementary 3), mapped possible causal relationships that were discussed across the research team and refined as CMO configurations. These guided the interviews with stakeholders and the theory testing review work in stage two.

### Stage 2: Candidate theories testing and refinement through further iterative searches

**Fig. 2:**
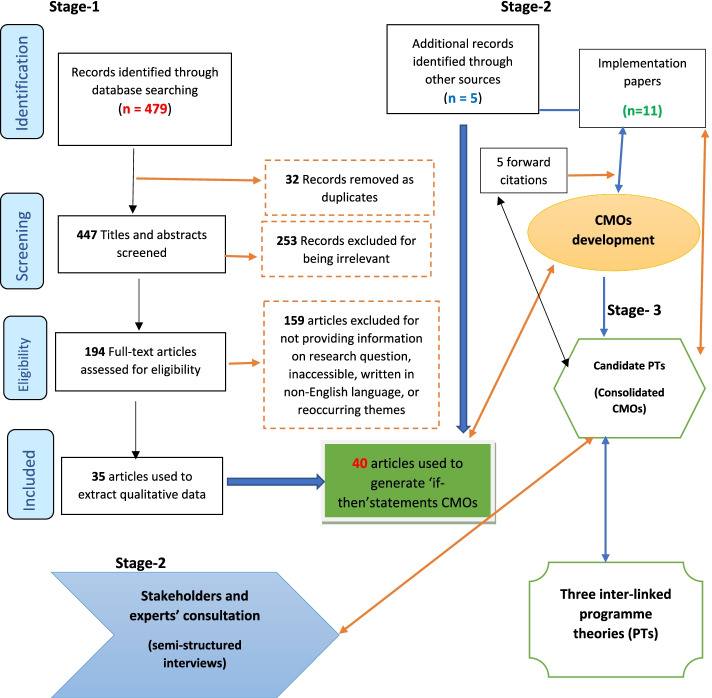
Document flow and review processes: conceptual diagram of database searches, snowballing searches, and iterative cycles

Based on the theoretical propositions derived from the scoping work, search terms were reviewed. The database searches in Stage 1 (described above) were extended with lateral searches and forward citations of relevant documents, to capture studies on digital engagement, care home cultures that support uptake of MDS and additional implementation studies (Fig. 2). A bespoke structured data extraction form, based on the CMOs, captured how the MDS/assessment tool was used at the resident level of care.

Interviews with stakeholders and discussions across the research team discussed how the theories resonated with them as experts and possible alternative explanations relevant for the successful development and use of an MDS.

### Stage 3: Data analysis and synthesis processes

**Fig. 3:**
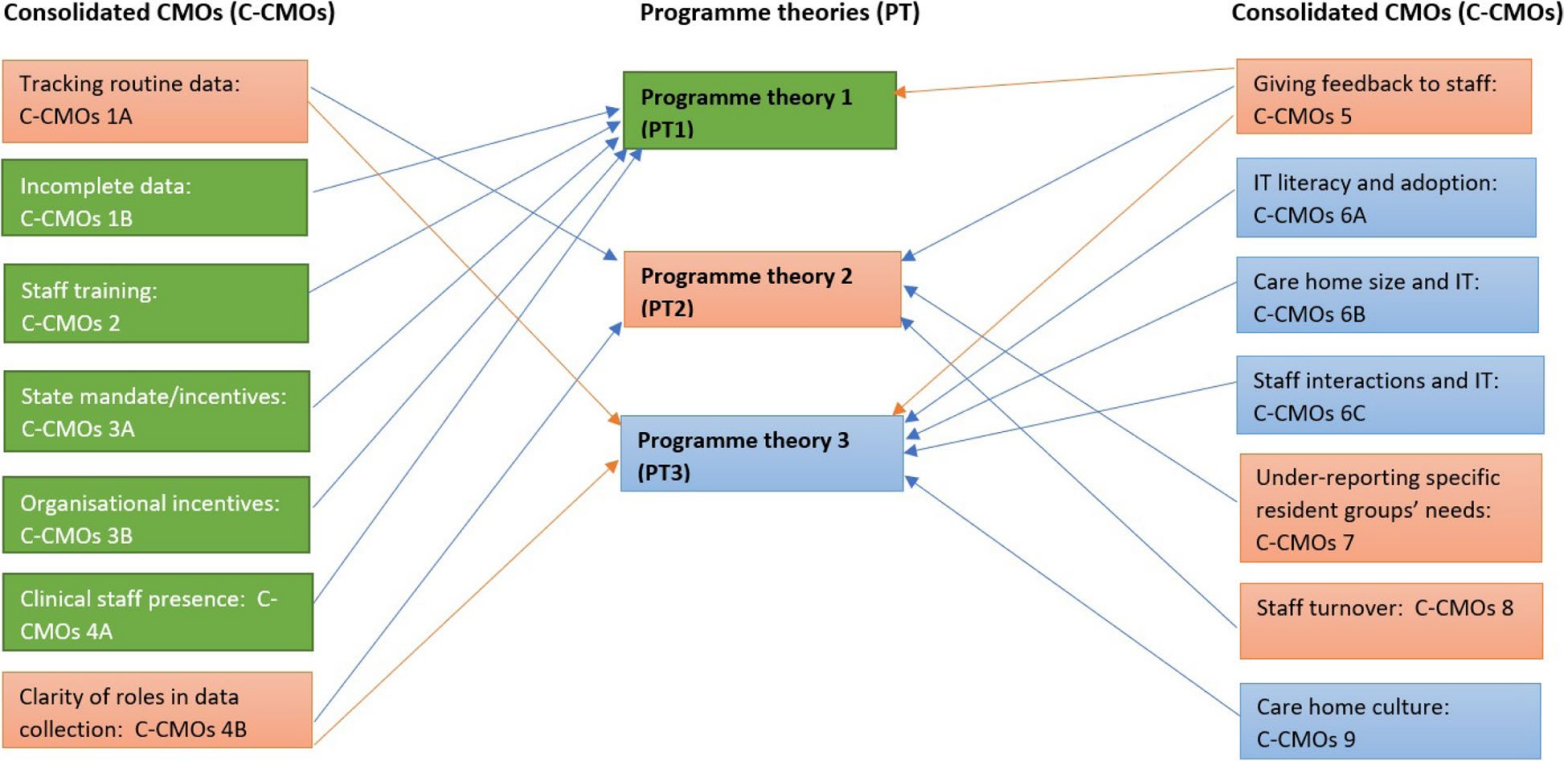
Programme theories codification process

**Fig. 4:**
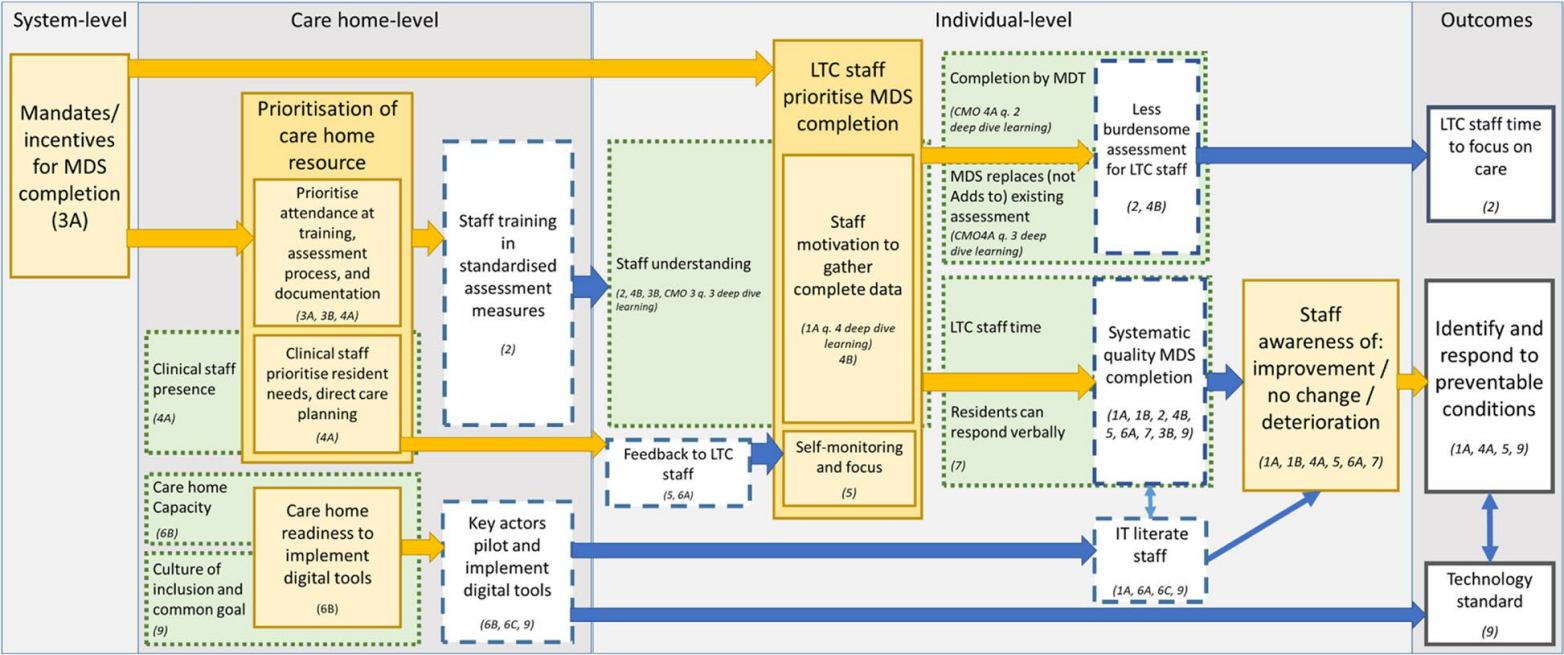
Mapping of CMOs and outcomes

Data analysis focused on how the evidence built upon, refuted or provided alternative explanations for the CMOs. First, observable patterns in context and outcome (demi-regularities) detectable within and among the data were reviewed and formed into a list of CMOs (Table 1). From this, three consolidated programme theories (PTs) were formed (Fig. 3) to explain how the use and uptake of MDS worked, for whom and in what circumstances. The way the CMOs were organised to capture how MDS may work at the organisational, staff and resident levels of care is presented in Fig. 4.

## Results

The findings are presented in linear way, the analyses however, involved iterative processes as shown in various figures containing double arrows.

### Characteristics of included studies

Of the 479 records initially retrieved from electronic database search, 194 were included for full-text screening (Fig. 2). Most papers that completed secondary data analysis of MDS were excluded because they did not provide any data on the use of MDS within the care home. Studies were included that had a population focus but also discussed the quality of reporting, and anomalies, for example for residents from different ethnic backgrounds [46–48] or in receipt of different types of funding [18]. Thirty-five papers were included from the search. Five additional publications were identified through lateral searches; 40 full papers were included in the scoping review (Fig. 2). Of these, over two thirds came from North America (USA (n=25), Canada (n= 7) with one or two papers from each of Australia (n=2), Taiwan (n=2), Italy (n=1), New Zealand (n=1), Norway (n=1), and the UK (n=1) (Online supplementary 4).

Thirty-three ‘if-then’ statements were formed from literature data (Online supplementary 3). Of these ‘if- then’ statements, 14 recurring themes in the data formed the basis of our consolidated CMO (C-CMO) configurations (Table 2). We then carried out further background literature, lateral and forward citations and theory driven searches for phase two and identified additional 16 references, 11 of which were papers on implementation research in care homes (Fig. 2). We finally codified the nine C-CMOs into three interlinking programme theories (Fig. 3).

**Table 2:**
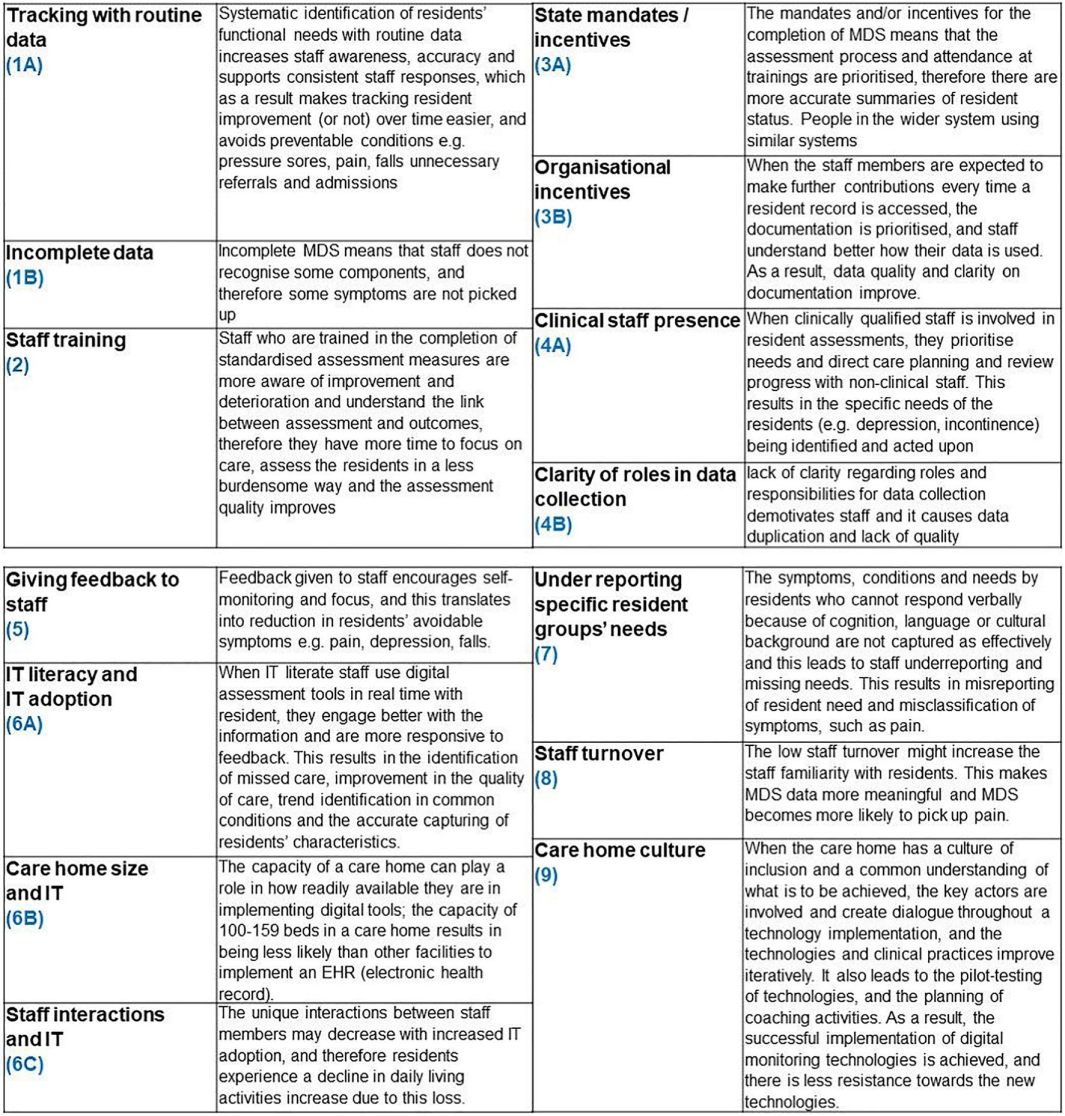
List of CMOs

### Stakeholder interviews

Each interview lasted 45 mins – 1 hour. Key issues identified from the interviews that informed the interpretation of the scoping review findings and final analysis were: feedback to staff on residents’ status; frequency of data capture in a working day; staff members’ pre-existing knowledge of the resident; consistency of care among staff and how this influences their ability use digital approaches to data capture. A recurring topic was how to resolve the need to complete MDS aggregated at the care home level, for example, for planning and audit and MDS use for individual residents’ daily care.

### Programme theory

Three key inter-linked theoretical propositions, based on nine consolidated CMOs (C-CMOs), articulate what supports the uptake and use of MDS by frontline staff (Fig. 3). These focused on how mandates and incentives, the involvement and oversight of clinicians in the use of MDS and staff skills and readiness to engage with digital technology led to meaningful data capture (or not) (Table 3).

**Table 3:** Three interlinked programme theories

**Motivation**: Mandates and incentives for MDS completion combined with MDS training and clinician involvement for care home staff motivates staff to complete MDS for residents and use this as the basis for discussion and care planning to identify residents’ needs and review care.	
**Frontline staff monitoring**: Completion of the MDS is built into the working practices, monitoring, and record systems of all staff (including visiting clinicians) involved in residents’ care, with junior staff contributing to data entry. This creates an accessible comprehensive account of residents’ needs, supports continuity of care, especially in instances of residents who cannot respond verbally because of cognition, language, or cultural background.	
**Embedded recording systems**: When staff MDS recording systems are embedded as part of the care home approach to collecting resident data and staff are skilled in using digitally based systems to record residents’ needs as part of a person-centred care process the accuracy and relevance of data will reflect residents’ experiences and be used as the basis for care planning and review and reduce time away from providing care	

The three key theoretical propositions (Box 2) are inter-linked. The programme theory showing how these relate to each other and to the desired outcome of MDs are shown in Fig. 4 (see online supplementary file 5 for a more detailed mapping of the contexts, mechanisms and outcomes involved in moving from mandates to outcomes). The main mechanism to achieve the identification of, and responsiveness to, preventable conditions in care home residents (O) was frontline staff awareness of residents ‘improvement or deterioration (M) (Fig. 4: C-CMOs 1A, 1B, 4A, 5, 6A, 7). This awareness (M) depended upon frontline staff prioritising MDS completion, which depended upon the prioritisation of care home resource to achieve MDS completion which in turn, depended upon a system-level mandate for MDS completion. There are three key pathways from mandate to MDS completion: care home staff motivation (key theoretical proposition 1), care home staff self-monitoring and focus (key theoretical proposition 2), and IT literate care home staff using digital assessment tools in real time with residents (key theoretical proposition 3) (Box 2).

It was unclear how care home staff prior knowledge of residents’ affected completion of an MDS. Low staff turnover for example, could either mean familiarity with residents, improved documentation of residents’ needs or that important changes or significant pieces of information were missed.

The three key theoretical propositions are described in detail below.

### Motivation

It is the motivation of frontline staff to gather complete data about residents that supports systematic and complete identification of residents' functional needs and ensures staff awareness of residents’ changing status. This is reliant on staff understanding the link between their assessment and resident outcomes, their roles and responsibilities for data collection, and how their collected data will be used (Fig. 4).

Theory development suggested that frontline staff motivation can be increased through training in the completion of MDS and purpose of MDS data (Table 1). Most of the evidence reviewed was from North America. Completion of MDS in these settings is mandated by the federal government or the state. It is not discretionary. Whilst there was a sense in which staff might feel ‘forced’ to complete an MDS due to care home prioritisation of training in response to system-level mandates, this ‘forcing’ [22] could lead to the sustained use of MDS. It enabled staff to discover the benefits and thereby develop critical individual-level motivation to use the MDS in everyday practice. Here, the motivation might come initially from external mandates, but is then internalised and sustained through the process of engaging with MDS. Motivation was adversely affected where there was underinvestment in training or training that did not involve staff who were providing direct care. If the MDS presented as an administrative task this took the focus away from the resident. There were examples of how this led to incomplete collection of data, or data completion with groups of residents’ needs not fully reported [49] [19, 25, 50]; (also, see Table 1).

Staff training by care homes depended upon prioritisation of team resource toward MDS completion. This included prioritising attendance at training, the assessment process (Fig. 4: C-CMO 3A), documentation (with staff being expected to edit resident records on each assessment; 3B), involvement in direct care planning, and review of frontline staff progress with clinical staff (which requires clinical staff in the care home at least some of the time; 4A).

Initial motivation to complete an MDS may rely on this training and care home level reinforcement of the utility of the data for improving resident care. In the long term, sustained motivation can develop through the feedback loop of staff witnessing changes in resident care due to systematic data collection and frontline staff having a shared confidence that their observations are valued. This may represent a critical aspect of the sustainability of MDS use in care homes. Completion of specific aspects of an MDS, e.g. continence and oral health care [51] illustrated how training affected staff engagement with an MDS. One review on MDS completion identified the needed interplay between competency in completing the MDS, training to support understanding of the categories of assessment, and review and staff engagement with managers and clinicians on residents’ behalf over time. On page 21, the authors note:

“*Data quality in the MDS will continue to reflect characteristics both of the instrument itself and of the assessors*, *their training and support*. …*Consequently, ongoing education of clinical staff and health managers with respect to assessment practices and applications of the MDS is important”* [52]. A plethora of evidence suggest the need for a range of training and support (Table 1), but it is unclear how some approaches provoked more lasting engagement than others. It may be fruitful for the sector to develop and test tools, such as frontline staff sharing groups, to further enable this feedback loop.

In respect of some residents however, the incentive or mandate accompanied with training might be insufficient to motivate sustained completion. For example, this might apply to people at the end of life, possibly because staff knew that residents’ deterioration was irreversible (Table 1).

The motivation of frontline care staff to complete resident data in a systematic way may result in less burdensome assessment (for example by reducing duplication) releasing more time for frontline care staff to focus on resident care (Table 1: C-CMOs 2 and 4B).

### Frontline staff monitoring

The process of MDS completion and personnel involved affect how residents’ needs are captured and the impact this has on the working of the care home. To collect resident data in a systematic way, frontline staff must monitor their data collection (Fig. 4: C-CMO 5). This self-reflexive monitoring is supported by receiving feedback by health care professionals. At the resident level of care, under-reporting (or omission) of information was linked to carers relying on their own judgement and interpretation of the intensity of residents’ experiences. This was evident when residents could not communicate their needs, for example in relation to pain and depression [23, 25, 53]. For an MDS to operate requires the regular involvement and engagement of health care professionals both within and from outside the care home (C-CMOs 6A) who are prioritising resident needs, direct care planning, discussing and reviewing progress with care staff (C-CMOs 4A, 4B, 5).

The MDS values standardising and creating a common language between different practitioners both visiting and caring for residents. There was an underlying tension about whose language and categorisation dominated. One stakeholder interview had described how the perceived medical orientation of the MDS affected the detail staff might record or how they would use it if their understanding of the resident did not fit with the categorisation. Key to effective monitoring is ensuring the MDS allows care home staff involved in direct care to share with others their observations and personal insights (e.g., change of shift reports, family conferences) [22, 54, 55]. Structured opportunities within the MDS to share this kind of information are an important context that respects and values the knowledge of all care staff and not just health care professionals. Studies of MDS implementation described that care staff and health care professionals needed to have ongoing conversations about case load organisation and the significance of what was being recorded to support ongoing engagement in using the MDS (Table 1).

Completing a typical MDS is time-consuming. Where IT literate staff are using digital assessments, this helps them to receive and respond to feedback from clinical staff, and thus to increase their self-monitoring and systematic completion of an MDS (Fig. 4: C-CMOs 1A, 5, 6A,6B,6C). The availability of designated staff with protected time to complete an MDS is compromised by staff shortages and turnover [56, 57] (also see Table 1). One early study found that the resident’s first assessment takes 60–90 minutes to complete [22]. Over two decades since that report, the time required is similar [23, 58–61]. This links to our first proposition and the need for training and resources to create a shared understanding and motivation to use the MDS as the basis for care.

### Embedded recording systems

Digital assessment tools used in real time with residents by IT literate staff seemed important for MDS completion (C-CMOs 6A, 6B, 6C, 9, 1A). Care homes with decreased uptake of health information technologies were theorised to experience decreased benefits from an MDS. The stakeholder interviews and scoping review suggested this went beyond a recognition that being comfortable with information technology (IT) affects uptake and use of an MDS. A quality manager of a care organisation argued when interviewed that most care staff (80-90 percent) wanted to provide care rather than spend time on technologies. There was little theory around this in the identified sources or further purposive searches of the literature. The theory developed in this review points to the importance of care-home level actors, usually care home staff, implementing digital tools in ways that create dialogue with all staff throughout piloting and implementing these tools (C-CMOs 6B, 6C, 9). When all care home staff were actively engaged with electronic documentation systems, the detail of residents’ care improved, for example, for residents experiencing incontinence [62, 63]**.** Equally, adoption of IT and losing the opportunity to discuss and receive information face to face about residents’ care affects whose information is included or excluded, and who can access (or not) information [55]. Balancing the preferences of staff and the use of IT at the point of care are important [63, 64].

Care home readiness to implement digital tools will also play a critical role (CCMO 96B), which will depend upon care home capacity (CCMO 6B) size and the culture of inclusion and of having a common goal (CCMO 9). When it was clear that integrating an IT based MDS into daily care routines supported care processes and benefitted residents, their families, and staff, there were improved resident outcomes in key areas; for example, activities of daily living and in residents physical activity [65]. How other sources of information and use of parallel systems of recording information (e.g. paper records) diminish will affect the embedding of an MDS and if it captures nursing home residents’ experience of care [20, 55]. A Belgian study found it took a year to integrate the InterRAI Palliative Care instrument into the day-to-day practices of a nursing home [23].

## Discussion

This review explains how the uptake and use of MDS may improve outcomes for staff and residents, and in what circumstances to provide an account of how an MDS can support residents’ care in settings with no prior history of its use.

From the outset we knew that mandates and incentives were a key context and that bottom-up and top-down approaches were needed to converge for effective and successful implementation [66]. Reform is more likely when policies are viewed as clinically relevant, coherent and achievable, then the regulations become more sustainable over time [67, 68].

Triggering of responses that lead to changes in residents’ assessment and care needed additional contexts; training for all members of staff on the significance of different assessment categories for direct care and ongoing involvement of clinicians in the assessment and review of resident data. These generate a sense of collective purpose, understanding and recognition of the value of using residents’ data to inform care. There are implications for how this is funded and if all care homes and their staff have equal access to this level of support and training. A key finding that appeared to be specific to the long-term care workforce, was the importance of staff confidence when both entering and using data, and if entering and reviewing resident data were perceived as separate to, and a distraction from, care work.

These findings resonates with the four domains of the Normalisation Process Theory (NPT) (collective action, coherence, cognitive participation and reflexive monitoring) and how meaningful change occurred when the relevant actors were persuaded that the new system would be as good if not better, in a context where the imperative to make a change was externally imposed [69]. The review demonstrated the human processes and responses involved when seeking to “normalise” the MDS into the work of the care home. Key, was investing in training that went beyond equipping staff to be skilled in data capture to enable a shared understanding (coherence) about why they would want to use information from an MDS when discussing residents and making decisions.

Advocates of an MDS argue that standardised forms of assessment provide a foundation for ongoing care, contributing to understanding residents’ needs and early identification of potential risks and problems. This is grounded in theories of multidisciplinary working and what supports continuity of care for older people with complex and varied needs based on evidence of what works [70–72]. Evidence of the impact of multidisciplinary team working in care homes demonstrate improvement in patient assessment and management practices, including responsive behaviours, falls, use of antipsychotics, depressive symptoms, appropriateness of medications, restraint use, nutrition, and pain [73–75]. For an MDS to be able to exploit these known benefits of multidisciplinary working, staff training should follow the same approach, with all types and grades of staff learning to use MDS together creating opportunities to contribute their different knowledge about the resident. Having a member of staff responsible for how an MDS was completed was important to ensure completion and protected staff time. However, without structured opportunities for everyone to contribute and residents’ priorities to be included, it could disenfranchise the knowledge of those giving and receiving the care and introduce a false divide between those who controlled the information and those who did not. As a resource, how MDS adoption and use are introduced links to social identity theories and research on what fosters a shared approach and agreement around goals and values for providing care in long term settings [76]. In long term care, staffs’ social identity and engagement, in this case with an MDS, needs to demonstrate how it meaningfully supports specific norms, and values about residents’ care. Interventions to encourage the uptake of an MDS that support identity mobilisation and working practices that can reinforce group relations are the drivers for uptake and change. Without this, involvement in an MDS risks being a distraction and threat to the groups’ values of how they define and value their care work. By valuing the contributions of staff and building on existing ways of working, the uptake and use of an MDS has the potential to enable all staff to learn with and from each other about what is important for residents and their care.

Despite the extensive use of MDS as a data source for commissioning and research, most papers that relied on these data were excluded, because of the absence of discussion in the papers about the quality of the data, how it was entered and learning about underrepresented groups and missing data.

The evidence in this review supported the importance of ongoing engagement of health professionals from outside of the care home, for example geriatricians [73, 77, 78]. The detail did not allow us to conclude if a mix or a particular professional group were needed to enable MDS uptake.

Care homes have characteristics that affect uptake and use of innovation [77–81]. At the resident level of care, we know care home staff have limited access to training, low pay, and a high turnover of employment [79, 82, 83]. There is little or no evidence of the efficacy of stand-alone care home staff training unless it is linked to mechanisms of ‘reinforcing’ (e.g., additional supervision or individual skills training), or ‘enabling’ (e.g., help to put learning into practice) [84]. Arguably, the daily use of the MDS, training and clinician engagement described in the few implementation studies retrieved could trigger these responses. There was, however, no agreement about how long was required to ensure engagement with an MDS and what ensured that staff involved in care habitually used the MDS. Nor was it discussed if the length of time between staff entering resident information and it influencing decisions about residents’ care affected how staff subsequently used the information for discussions and feedback to staff on resident outcomes. Claims that staff could be supported to use the MDS in a few sessions do not fit with studies that have required cross care home engagement and staff participation to change practice [85–87]. Research testing theories of goal setting has demonstrated that communications in care homes can be improved by providing feedback, guided by goal-setting theory and that highly resource-intensive feedback interventions may be unnecessary [54]. To be effective however, this would need an individual’s goals to include gaining skills in using an MDS and organisational support.

The challenges of IT implementation are well documented, but this review raised questions if characteristics of the care home workforce and the care home location in relation to other systems of care affected uptake and use of MDS. The relationship between nursing, senior and junior care staff within the care home, prior experience, level of anxiety about IT and type of responsibilities, are issues identified by other authors in other mixed care settings as affecting staff engagement [88]**.** Depending on who was confident enough to use MDS and the supporting technology, who had permission to use it and opportunity to inform the MDS either created a sense of shared endeavour or led to parallel systems of information exchange for the purposes of care. Linked to this was how resident data could be shared to inform care with outside organisations. COVID-19 in the UK has exposed the difficulties of linking data on residents to inform decision making and the need for digital integration is a recognised priority [8–10].

The proposed programme theory is constrained by the evidence that was available and the inferences that could be made from the data. Available evidence clustered around the training for staff, their actions and organisational support. There was less evidence on how MDS use facilitated communication within the care home and linked outcomes at the resident level of care.

### Strengths, limitations, and future research directions

This review asks how MDS can be implemented in care home settings. The strength of realist approaches is the focus on how different contexts can generate different responses from participants and so outcomes. It is a strength of this review that it articulates the contexts and care home specific mechanisms that are likely to lead to uptake of MDS at the resident level of care.

Most of the evidence was North American, and the organisation and structure of the surrounding systems of health care were not explored in this review. A study in England argued that recognising the relationships care homes have with other providers, and the wraparound care received from external health care providers, directly affected residents’ experience of care and access to medical support [89].

There is no evidence presented in this review about the wider narratives and discourses around nationally deployed social care information systems and/or minimum data sets. Greenhalgh and colleagues suggest that for the application of technologies, creating an effective inter-stakeholder dialogue and building learning communities are necessary in realising a focal community idea [90]. This could be relevant in addressing our finding about what needs to be in place for the introduction of electronic forms of minimum data sets in the care sector at a wider level. The learning from the pandemic has meant that there is a greater openness to discussing change and how to share information within and across health and social care.

## Conclusion

Research has demonstrated the value of MDS to commissioners and service providers in the identification of care needs. This review focused on how its use on a day-to-day basis could influence care work and resident outcomes. A national/federal mandate is highly relevant for the success of an MDS, but not always meaningful or beneficial to the staff who provide care. If, however, it is implemented with training and clinician engagement, its use can be a key motivator for improving day to day resident care and outcomes, as well as regional and national understanding of the care home population.

This analysis enables us to articulate how data informed discussions about residents can be normalised by focusing on the working environment of the care home and the way in which an MDS is introduced, discussed and used over time. It directs attention to the important issue of how to tailor and implement an MDS likely to inform residents’ everyday care, by identifying the causal mechanisms of, prioritising data capture, staff and clinician engagement, and staff confidence and the contexts that enable them. Achieving this requires resources: funding and time to support staff training and strategies that sustain engagement and motivation from staff and visiting practitioners. This will ensure that resident data in an MDS are valid and valued by care home staff as an aid for care rather than an administrative burden.

## Supplementary Information

**Additional file 1.** Interview schedules.

**Additional file 2.** Example of search terms used across databases to retrieve relevant literature.

**Additional file 3.** If-then statements.

**Additional file 4.** Sources of papers per countries.

**Additional file 5.** In-depth programme theory diagram to supplement Fig. 4.

## Data Availability

The data generated and analysed during the current review are not suitable for sharing beyond that contained within the report. Further information can be obtained from the corresponding author on reasonable request.

## Abbreviations

CMO: Context Mechanism Outcome
InterRAI: LTCF: International Resident Assessment Instrument Long Term Care Facility
MDS: Minimum Data Set
