# Supplementary material for: The uptake and use of a minimum data set (MDS) for older people living and dying in care homes: a realist review
Source: BMC Geriatr. 2022 Jan 7;22:33. doi: 10.1186/s12877-021-02705-w (PMC8739629; doi:10.1186/s12877-021-02705-w)
Supplement: Supplementary file 1 — Additional file 1. Interview schedules. [file 12877_2021_2705_MOESM1_ESM.docx]

**Online supplementary 1: Interview schedules**

More general questions

1. What is (was) your role in the care home?
2. Do you use paper or electronic records (or both)?
3. Do you record or access information related with resident’s wellbeing and health on a regular basis? In what situations do you use them?
4. Is the data in your care home linked with other health and social care systems?
5. What are your perceptions about the value of using a comprehensive minimum data set in care homes?
6. How do you think care staff (both clinically qualified and other non-qualified) use the data they collect, and how does the data inform the care they give day by day?
7. Are there any challenges related with completing assessment forms and notes about residents?
8. Do you think there are some residents who have less information recorded about (if so, why?)
9. Is there anything you would like to change about the system?

Prompts to link CMOs/if-then statements

1. There is bidirectional theory that says knowing a resident very well may have impact on the level of information that is recorded about that person. In your experience, what do you know? (CMO 1A – Tracking routine data; also linked to 1B – incomplete data).
2. How long does it require for the person(s) involved in assessing a resident takes to complete an MDS successfully and efficiently?
3. What is the relationship between MDS data entry clerks and decision-makers in care planning?
4. How staff understand their responsibilities in recording care home data?
5. What is it that forces/encourages staff to record what they do? Is it because of the presence of senior staff, clinicians, or is it because it is mandated?
