# Supplementary material for: The uptake and use of a minimum data set (MDS) for older people living and dying in care homes: a realist review
Source: BMC Geriatr. 2022 Jan 7;22:33. doi: 10.1186/s12877-021-02705-w (PMC8739629; doi:10.1186/s12877-021-02705-w)
Supplement: Supplementary file 2 — Additional file 2. Example of search terms used across databases to retrieve relevant literature. [file 12877_2021_2705_MOESM2_ESM.docx]

**Online supplementary 2: Example of search terms used across databases to retrieve relevant literature**

| 1. *Homes for the Aged/ |  |  |
| --- | --- | --- |
| 2. *Nursing Homes/ |  |  |
| 3. *Long-Term Care/ |  |  |
| 4. *Residential Facilities/ |  |  |
| 5. *Respite Care/ |  |  |
| 6. *Intermediate Care/ |  |  |
| 7. "care home$".ab,ti. |  |  |
| 8. "nursing home$".ab,ti. |  |  |
| 9. "residential care".ab,ti. |  |  |
| 10. ("long term care" or "long-term care" or "longterm care").ab,ti. |  |  |
| 11. "home$ for the aged".ab,ti. |  |  |
| 12. "care facilit*".ab,ti. |  |  |
| 13. "old$ people$ home$".ti,ab. |  |  |
| 14. (retir$ adj2 home$).ab,ti. |  |  |
| 15. ("old$ adult$" adj3 (facilit$ or residential or accommodation)).ab,ti. |  |  |
| 16. ("old$ people$" adj3 (facilit$ or residential or accommodation)).ab,ti. |  |  |
| 17. ("old$ person$" adj3 (facilit$ or residential or accommodation)).ab,ti. |  |  |
| 18. ((geriatric$ or elder$ or senior$ or retir$) adj3 (facilit$ or residential or accommodation)).ab,ti. |  |  |
| 19. "respite care".ti,ab. |  |  |
| 20. "intermediate care".ti,ab. |  |  |
| 21. or/1-20 |  |  |
| 22. *Randomized Controlled Trials as Topic/ |  |  |
| 23. Randomized controlled trial/ |  |  |
| 24. Random allocation/ |  |  |
| 25. Double blind method/ |  |  |
| 26. Single blind method/ |  |  |
| 27. Clinical Trial/ |  |  |
| 28. Clinical trials as Topic/ |  |  |
| 29. "randomi*ed".ab,ti. |  |  |
| 30. randomly.ab,ti. |  |  |
| 31. controlled clinical trial.pt. |  |  |
| 32. Evaluation Study/ |  |  |
| 33. Comparative Study/ |  |  |
| 34. "before and after study".ti,ab,mp. |  |  |
| 35. or/22-34 |  |  |
| 36. "Outcome and Process Assessment (Health Care)"/ |  |  |
| 37. *Implementation Science/ |  |  |
| 38. "process evaluation".ab,ti. |  |  |
| 39. (process$ adj3 evaluation$).ab,ti. |  |  |
| 40. (program$ adj3 evaluation$).ab,ti. |  |  |
| 41. implementation.ab,ti. |  |  |
| 42. context$.ab,ti. |  |  |
| 43. fidelity.ab,ti. |  |  |
| 44. or/36-43 |  |  |
| 45. *Qualitative Research/ |  |  |
| 46. *Focus Groups/ |  |  |
| 47. *Interviews as Topic/ |  |  |
| 48. *Narration/ |  |  |
| 49. (("semi-structured" or semistructured or unstructured or informal or "in-depth" or "indepth" or "face to face" or structured or guide) adj3 (interview$ or discussion$ or questionnaire$)).ab,ti. |  |  |
| 50. or/45-49 |  |  |
| 51. 44 or 50 |  |  |
| 52. 21 and 35 |  |  |
| 53. 51 and 52 |  |  |
| 54. limit 52 to yr="2009 -Current" |  |  |
| 55. 53 or 54 |  |  |
