# Supplementary material for: The uptake and use of a minimum data set (MDS) for older people living and dying in care homes: a realist review
Source: BMC Geriatr. 2022 Jan 7;22:33. doi: 10.1186/s12877-021-02705-w (PMC8739629; doi:10.1186/s12877-021-02705-w)
Supplement: Supplementary file 3 — Additional file 3. If-then statements. [file 12877_2021_2705_MOESM3_ESM.docx]

**Online supplementary 3: If-then statements**

| **References** | **Training or education** | **Skills of who input data** | **Residents’ characteristics** | **Common language** | **incentives** | **Resources** |
| --- | --- | --- | --- | --- | --- | --- |
| **Onder et al (SHELTER Study) 2012** | If staff are trained about concepts of comprehensive geriatric assessment and multidisciplinary teamwork, then staff are likely to embrace an MDS.  If staff involved in assessment are trained and given enough time to carry out assessment, then components of an MDS are likely to be completed properly.  If training is provided to staff and there is access to readily use IT facilities, then staff resistance regarding use of an MDS may be minimised. | If implementation of integrated care is coordinated and carried out in step-by-step case management, then an MDS may lead to a positive impact on care providers and an MDT. | If an MDS contain uniform outcome measures that specifically address residents’ characteristics (e.g. ADL, CPS & DRS), then individual resident needs may easily be assessed and/or compared with other residents across different CH facilities. | If there is a common language among health and social care staff, then communication may improve among staff and stakeholders, thus leading to improvement in quality and continuity of care of CH residents. |  |  |
| **Vanneste and Declercq 2014** | If flexibility is introduced in training staff to use an MDS, then knowledge can be acquired easily, and skills applied appropriately. |  |  | If there is constant communication and collaboration between researchers and stakeholders, then the development of an MDS can suffice. |  |  |
| **Hansebo 1998** | If staff understand that the use of an MDS makes their work easier, then staff will be encouraged to use an MDS.  If there is a systematic use of an MDS (i.e. entry of accurate information), then it may improve staff knowledge and skills in working with elderly people. |  | If the MDT place the patient at the centre of care, then patient’s care is likely to be delivered uniformly over time.  If patient’s documentation is centralised, then it helps staff members to easily find and read about patient’s care plan. | If information to be entered in an MDS is concise and easily understood by its users, then communication across multidiscipline can become effective. | If there is incentive for training such as CPD credits, then staff will be motivated to attend MDS training courses. | If relatives are involved in patient’s assessment, then a holistic assessment of a patient may be completed, and the interaction may foster staff-relatives relationships. |
| **Doupe et al. 2018** |  | If healthcare use is accurately and timely entered in an MDS, then its output will reflect the true account of healthcare use at any given time.  If an MDS contain a software system that auto-populate with responses from previous assessment, then staff entering data on to an MDS must ensure that auto-responses of the system is either turned off, or the staff should manually make correction to avoid false positives in the MDS. |  |  |  |  |
| **Tran et al. 2019** |  |  | If the resident bed-days are not quality adjusted, then the low-cost nursing homes with low-quality services will appear to be more efficient, and this will affect the high-quality care in other care homes, because they will be seen as 'less efficient' and therefore will be forced to change how they provide care. |  |  | If efficiency is calculated without sound adjustments (that ignore quality), the quality of services given to residents may deteriorate due to laying off or not employing necessary skilled staff. |
| **Stampa et al. 2018** | If the clinical significance of routine use of MDS is not communicated to the care home staff from the beginning, then the assessment tool will be seen as a research tool by staff. | If the MDS is implemented in a setting for the first time, the staff may show resistance due to the fears of losing their professional autonomy. |  | If the standardisation of assessments is possible, it will allow managers to standardise practices and therefore this will lead to less confusion in the interpretation of MDS items. | If the MDS assessment tools are used at a national level, it creates a common language for all stakeholders. | If the care planning procedure is non-systematic, there will be higher resource utilisation and more hospitalisations from LTCF.  If managers and decision makers are not involved in the development of an MDS, this can pose a barrier in creating this common language.  If geriatricians are involved in the development of an MDS, it reinforces the leadership of a national MDS project. |
| **Dosa et al. 2006** |  |  |  |  | If the completion of RAPs is mandated, this can produce better quality in interpretation of MDS data. |  |
| **Devriendt et al, 2013** | If there is a guidance manual for the MDS instrument, then staff's data entry can improve in quality.  If the client has no previous MDS assessment, the staff may lack insight when conducting the first assessment, and therefore more errors in data entry may arise.  If the staff is not trained on software use, this can hinder the assessment process | If the assessment process is not managed well, the quality of data entry may deteriorate. |  |  | If funding for the staff conducting MDS assessments is not specifically dedicated, then the assessment process may not be efficient. | If the assessment personnel are overloaded with assessments, the quality of data entry may deteriorate. |
