# Supplementary figures and images for: The uptake and use of a minimum data set (MDS) for older people living and dying in care homes: a realist review
Source: BMC Geriatr. 2022 Jan 7;22:33. doi: 10.1186/s12877-021-02705-w (PMC8739629; doi:10.1186/s12877-021-02705-w)

Online supplementary 4


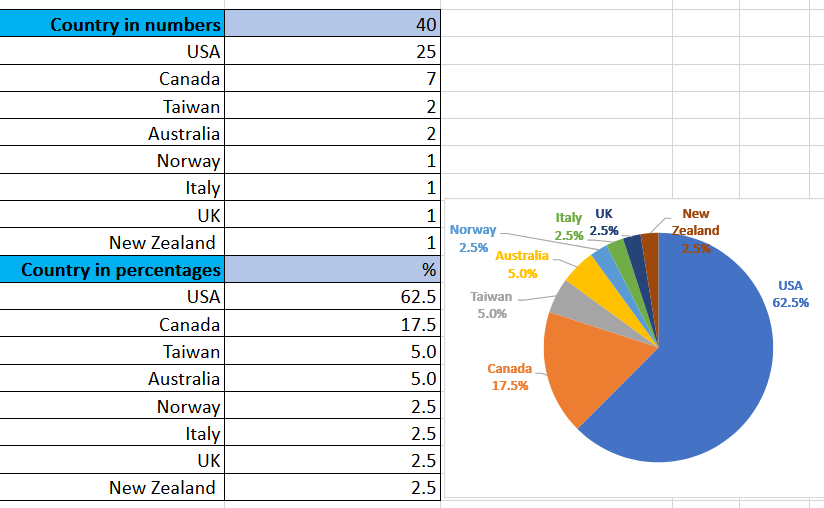

Supplement: Supplementary file 4 — Additional file 4. Sources of papers per countries. [file 12877_2021_2705_MOESM4_ESM.docx]

Online supplementary 5


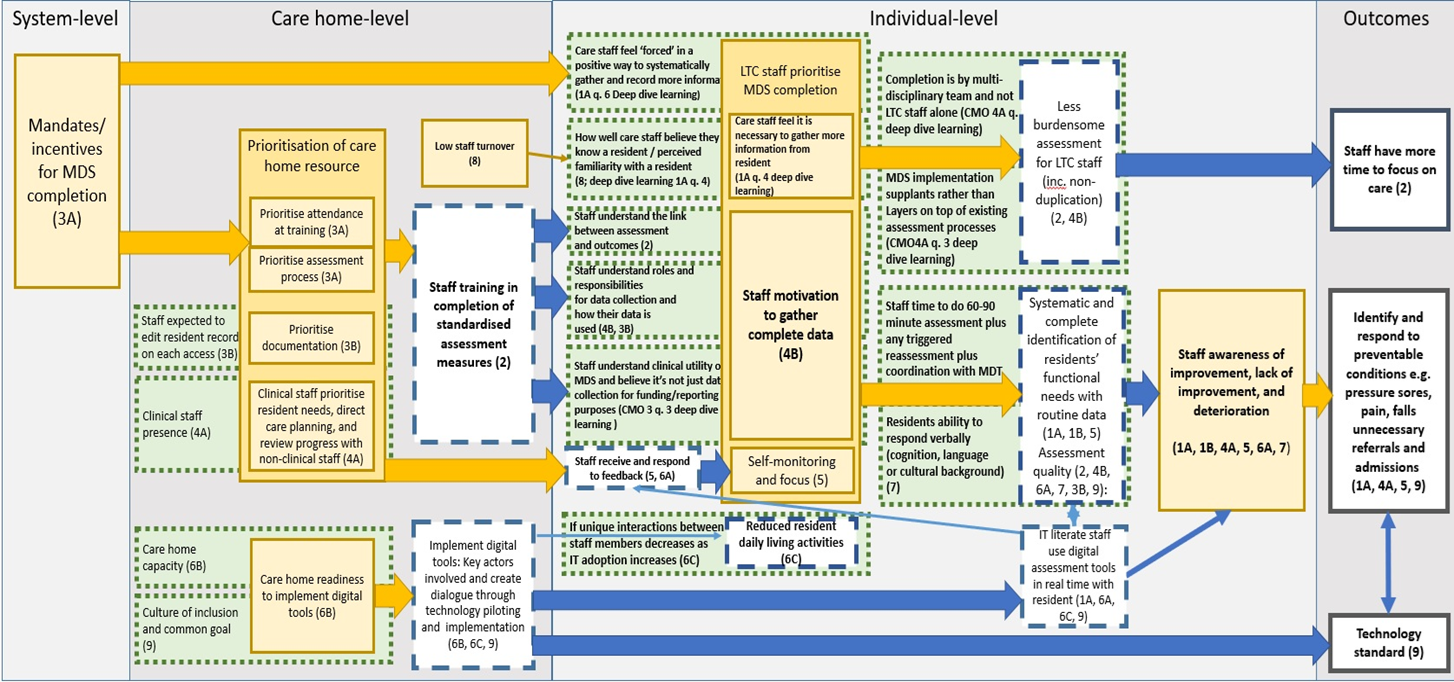

Supplement: Supplementary file 5 — Additional file 5. In-depth programme theory diagram to supplement Fig. 4. [file 12877_2021_2705_MOESM5_ESM.docx]
